# Bidirectional predictive modeling of narcissists’ social exclusion and their hostile interpretations: a deep learning-based exploration of cognitive mechanisms

**DOI:** 10.3389/fpsyg.2026.1761090

**Published:** 2026-03-05

**Authors:** Xiu Li, Xin Liu

**Affiliations:** 1 Department of Computer Science, School of Information Science and Technology, Xizang University, Lhasa, China; 2 Department of Development and Educational Psychology (Learning and Personality Development), Liaocheng University Dongchang College, Liaocheng, China

**Keywords:** hostile interpretation, multidimensional variables, bidirectional inference paths, deep learning, data-driven

## Abstract

This study aimed to clarify the interaction mechanism among hostile interpretation, narcissistic personality traits, and social exclusion/acceptance situations, address the limitation of traditional linear analysis, construct an accurate “trait-situation-cognition” prediction model (Hostile Interpretation & Bidirectional Prediction Network, HIBPN), and verify the intervention effect of self-affirmation on social exclusion. Two sequential experiments were designed: Experiment 1 enrolled undergraduate and postgraduate students, divided them by narcissistic traits into dominant narcissism, implicit narcissism, and neutral control groups, manipulated situations via Cyberball 5.0, quantified hostile interpretation biases, and validated situational effectiveness to provide modeling data. Experiment 2 selected implicit narcissists from Experiment 1, randomly assigning them to the self-affirmation intervention group (completing a value- and trait-oriented self-affirmation task) and control group (completing a neutral task). Consistent manipulation and tools with Experiment 1 were used to verify the intervention effect. The model integrated situational variables and narcissistic traits, establishing a closed-loop “antecedent-social exclusion-cognition” system and a bidirectional inference structure (the forward chain predicts social exclusion probability, and the reverse chain deduces the path of hostile interpretation bias). Results: In Experiment 1, under the 1:9 training-test set ratio, the predictive efficacies of the model’s two paths reached 69.9 and 83.2%, confirming significant interactions among the three variables; narcissistic traits accounted for 33.55 and 75.73% of the weights, serving as the core driving variable. In Experiment 2, preventive self-affirmation prior to social exclusion significantly reduced implicit narcissists’ hostile interpretation bias, with their subjectively predicted social acceptance rate 5.56% higher than that of the control group. This study reveals the dynamic interaction mechanism via HIBPN, providing a new cognitive prediction framework, and confirms the intervention value of preventive self-affirmation, offering a feasible approach to maintaining mental health.

## 1 Introduction

Hostile interpretations, one of the cognitive antecedents of aggressive behaviors (Quan et al., 2021), and their dynamic interaction mechanisms with narcissistic personality traits and the social exclusion still lack systematic modeling. Most existing studies rely on traditional linear regression or correlation analyses (e.g., Dyduch-Hazar et al., 2019), which are limited in capturing nonlinear interaction effects among multiple factors. For example, hostile attribution bias and anger immersion act as sequential mediators between trait anger and reactive aggression (Quan et al., 2021), while moderating the effects of gender and age (Zhen et al., 2011), further illustrating the complexity of individual differences. At the theoretical level, the concept of “narcissistic anger” was proposed in 1972, laying the foundation for understanding the association between narcissism and cognitive bias. Subsequent studies have confirmed a significant positive correlation between narcissism and hostile perceptions (Freund et al., 2022).

Specifically, individuals with vulnerable narcissistic traits depend on external validation and tend to interpret their environment negatively through hostile attribution biases (Zeigler-Hill et al., 2008; Miller et al., 2011)—a cognitive pattern that aligns with the core connotation of hostile interpretations. For grandiose narcissists, their inherent traits of superiority and exploitativeness (Pincus et al., 2014) drive narcissistic rivalry in interpersonal interactions, such as derogating others or holding negative perceptions of close relationships (Lamkin et al., 2014). This essentially reflects a hostile interpretive tendency: they tend to frame others’ neutral behaviors as threats to their own superiority. A meta-analytic review further corroborates this stable association: Urbonaviciute and Hepper (2020) found that both grandiose and implicit narcissism are significantly linked to reduced cognitive and affective empathy. The deficit in empathy directly impairs individuals’ ability to accurately perceive others’ intentions, thereby elevating the likelihood of hostile interpretations. Additionally, Zeigler-Hill et al. (2008) noted that narcissists’ self-worth is contingent on external approval. When confronted with ambiguous social feedback, they often adopt hostile attributions to safeguard their fragile self-identity, forming a cognitive chain of “ambiguous cues → threat perception → hostile interpretation.”

Notably, such hostile interpretive tendencies further shape narcissists’ perception of social exclusion: individuals who habitually adopt hostile attributions are more likely to perceive neutral or ambiguous social interactions as social exclusion (Zeigler-Hill et al., 2008). Conversely, perceived social exclusion reinforces their defensive motives to protect self-identity, forming a reciprocal cycle between hostile interpretations and social exclusion perception that is intertwined with narcissistic traits.

To situate this study within the broader field of social cognition and personality research, we draw on Bandura’s social cognitive theory (Bandura and National Institute of Mental Health, 1986), which emphasizes reciprocal determinism—the dynamic interaction between individual traits, cognitive processes, and environmental factors. This foundational theory has continued to exert profound influence across multiple psychological disciplines, with recent scholarly work further expanding its applications and validating its enduring relevance (de la Fuente et al., 2023). Specifically, de la Fuente et al. (2023) edited a research topic dedicated to Bandura’s legacy, compiling empirical evidence and extended models derived from social cognitive theory—including advancements in self-regulation, self-efficacy, and moral disengagement—across educational, developmental, and clinical contexts. Their work underscores that the core tenets of social cognitive theory, such as the interdependence of personal factors, cognition, and environment, remain pivotal for understanding complex psychological processes, aligning with our focus on modeling bidirectional interactions among narcissism, hostile interpretations, and social contexts.

Although Bandura’s theory provides a framework for understanding the dynamic interactions among the three factors, existing studies are still limited by traditional analytical methods when quantifying such complex relationships—and this limitation is not confined to research based on Bandura’s theory; scholars in other related studies have also faced the same issue. To date, most of them have employed statistical methods (e.g., Dyduch-Hazar et al., 2019) to examine the association between narcissism and aggression. However, statistical and traditional machine learning methods (e.g., random forests, decision trees) remain insufficient for capturing the complexity of these interactions.

**Figure 1:**
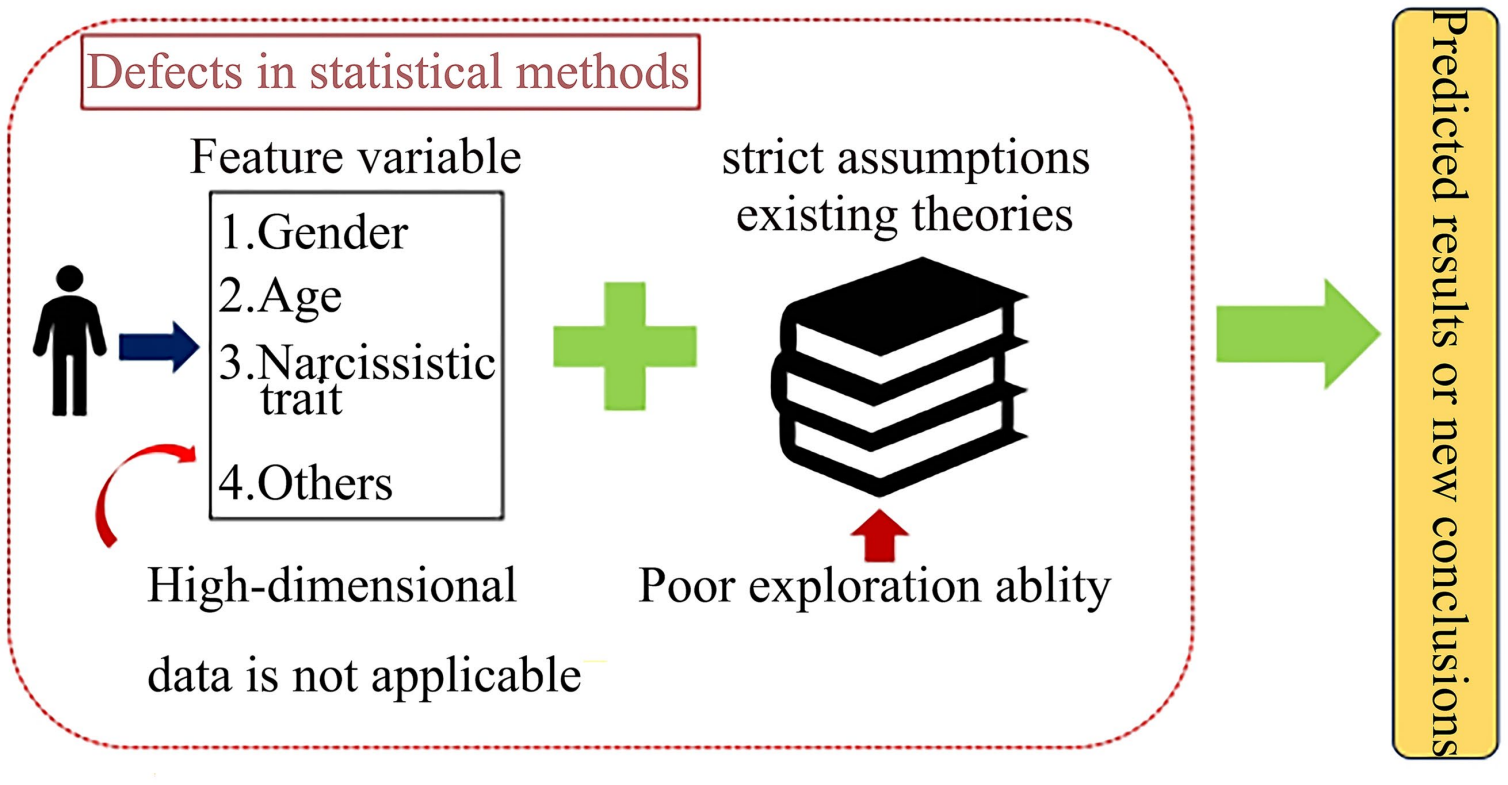
Schematic illustration of traditional statistical methods’ limitations—failing to capture nonlinear, high-dimensional interactions among narcissism, hostile interpretations, and the social exclusion, with higher inference errors and lower prediction accuracy than machine learning.

Statistical methods are highly dependent on strict assumptions or pre-existing theories, collect data at a superficial level without detecting the true psychological state of the subject, and are unable to delve into studies involving higher-dimensional variables (Cohen et al., 2003; Pearl, 2009) (Figure 1). They show clear limitations in exploratory analysis and in portraying the associations of psychological phenomena (e.g., insufficient ability to model nonlinear relationships and higher-order interactions). Wang et al. (2012) found traditional regression analyses struggle with complex psychological indicators (e.g., IVAR) and nonlinear data due to small sample constraints and model rigidity, causing notable statistical inference biases.

**Figure 2:**
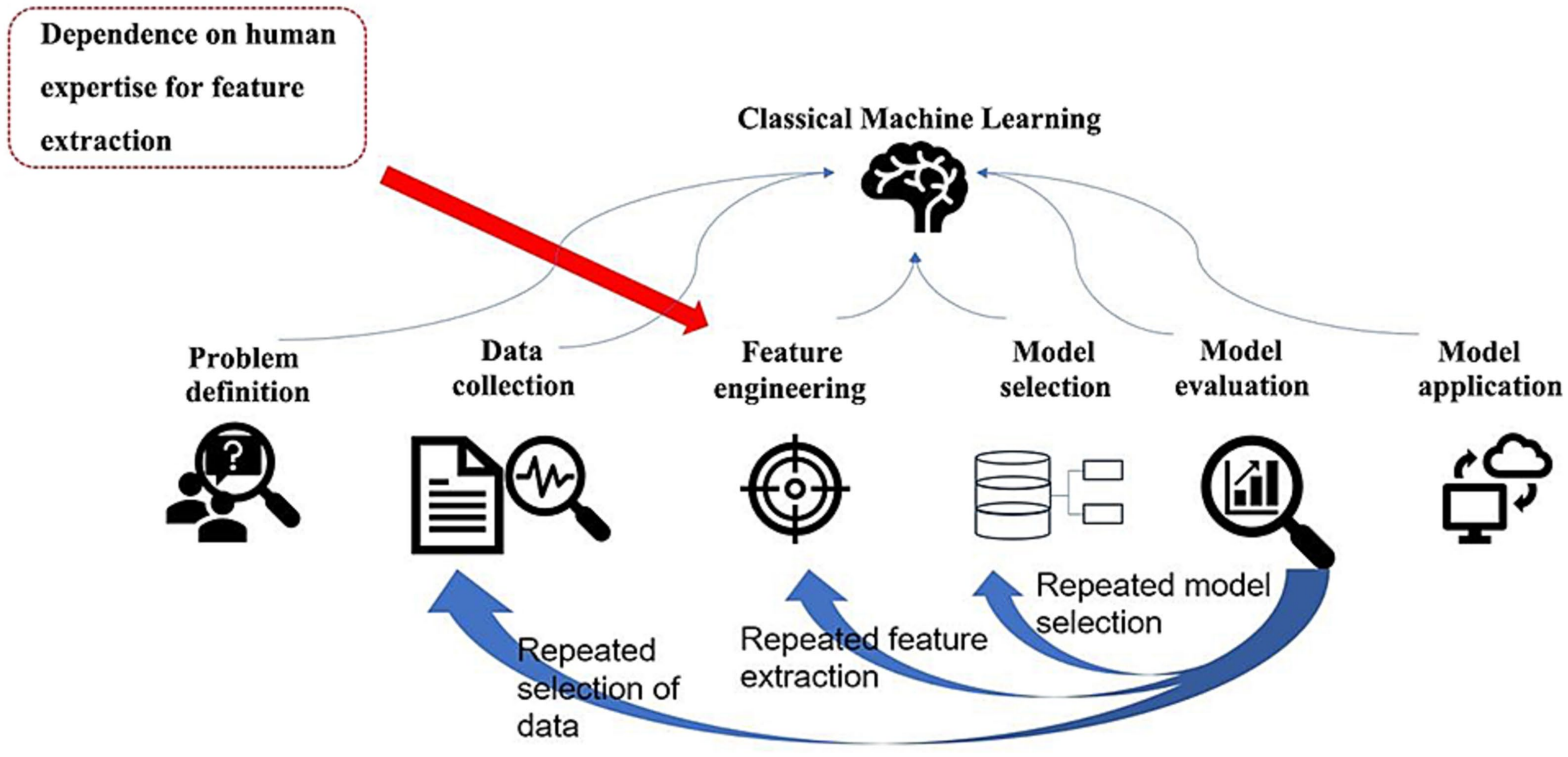
Classical machine learning workflow: It covers problem definition, repeated data collection, feature engineering (reliant on human expertise), model selection/evaluation, and application, with iterative loops between stages.

Traditional machine learning methods (e.g., decision trees, random forests) require manual feature engineering and still rely on a posteriori explanatory tools in complex scenarios (e.g., multifactor interactions) with limited granularity to explain the association paths between potential mediator variables, independent variables, and dependent variables (Hastie et al., 2009; Goodfellow et al., 2016) (Figure 2).

To achieve methodological innovation, break through the current research bottleneck in multifactor interaction analysis, and overcome the limitations of traditional linear approaches, this study developed the Hostile Interpretation and Bidirectional Prediction Network (HIBPN). Employing a multilayer perceptron architecture, HIBPN quantifies variables including narcissistic personality, hostile interpretation bias, and social context to model the complex mapping from psychological perception to behavioral output, thereby enabling bidirectional prediction of both social exclusion status and hostile interpretation bias.

The core innovation of this network lies in its dual theoretical anchoring. First, its modular design constitutes a direct operationalization of Bandura’s triadic reciprocal determinism (Bandura and National Institute of Mental Health, 1986): the “Human Characteristics and Environment Module” corresponds to environmental factors and personal traits; the “Narcissistic Score Module” corresponds to cognitive-affective factors (e.g., grandiose vs. vulnerable narcissism); and the “Hostile Interpretation Module” corresponds to behavioral-cognitive outcomes. Through dynamic interactions among these modules, the model instantaneously captures the mutual interdependence of person, cognition, and environment. Second, the bidirectional output function precisely operationalizes the “state-context bidirectional coupling” central to dynamic systems theory (Thelen and Smith, 1994a). The forward inference chain simulates the active construction of environmental perception (social exclusion) from cognitive states (narcissism), while the reverse chain models the feedback reinforcement of cognitive states (hostile interpretation bias) by environmental perception. This design replicates the dynamic equilibrium formed through continuous interaction (Thelen and Smith, 1994b)—for instance, the reverse pathway reveals how implicit narcissists’ hostile interpretations may reflect hypersensitivity to microsocial exclusion even in neutral contexts (Hansen-Brown and Freis, 2021). By transcending unidirectional causal assumptions, HIBPN inspired by recent applications of bidirectional recurrent neural networks in predicting cognitive-situational cycles (e.g., in anxiety disorders; Jiao, 2024).

This study employs ablation and intervention experiments to validate the model, offering a computational perspective on the cognitive mechanisms underlying aggressive behavior. The following sections detail the construction of HIBPN and present empirical validation results.

We asked the following three questions:

- Can deep learning accomplish the prediction of the social exclusion in which humans live under multifactor interaction conditions?
- Can it retain considerable generalization while performing prediction under these conditions?
- Can deep learning reveal the statistical association path between narcissism and social hostility interpretations by quantifying social exclusion states?

## 2 HIBPN model design

### 2.1 Modeling overview

To address the limitations of existing methods, we constructed HIBPN—a multilayer perceptron (MLP) based model for bidirectional prediction in the context of social interaction and cognitive bias. Following the methodological framework of Wang et al. (2024), we adopted a training-to-test ratio of 1:9 for model validation and selected the cross-entropy loss function to optimize classification performance. The bidirectional prediction mechanism operates as follows: first, the Hostile Interpretation Module, in combination with the Narcissistic Score Module and partial inputs from the Human Characteristics and Environment Module, predicts whether an individual is experiencing social exclusion or acceptance. Second, the Human Characteristics and Environment Module and the Narcissistic Score Module jointly predict the output of the Hostile Interpretation Module (i.e., the presence of hostile interpretation bias). The technical specifications of each module are detailed below. The mathematical formulations of the upscaling, downscaling, feature fusion, and prediction processes in each module are detailed in Equations (1–14) below.

### 2.2 Human characteristics and environment module

The Human Characteristics and Environment Module collects data on gender, age, type of environmental context (social acceptance or social exclusion), basic needs, estimated catch percentage, and personality type (dominant narcissism or implicit narcissism).

After collecting both human features and environment-related factors and considering the dataset size, we use a multilayer perceptron (MLP) with two layers to first upscale and then downscale the human features. Specifically, let $X_{h-e}$ denote the original input of human-environment features (e.g., gender, narcissism type, social exclusion context). The upscaling step maps these low-dimensional raw features to a high-dimensional space while preserving interaction information between individual traits and situational variables, formulated as:


$$Z_{h-e}^{\left(1\right)}=W_{h-e}^{\left(1\right)}\cdot X_{h-e}+b_{h-e}^{\left(1\right)}$$ (1)

$$A_{h-e}^{\left(1\right)}=\sigma (Z_{h-e}^{\left(1\right)})$$ (2)

Where $W_{h-e}^{\left(1\right)}$ and $b_{h-e}^{\left(1\right)}$ are the weight matrix and bias term of the first MLP layer, respectively, and σ(·) is an activation function (e.g., ReLU) introducing nonlinearity. $A_{h-e}^{\left(1\right)}$ represents the high-dimensional feature representation after upscaling.

Subsequently, the downscaling step retains core features while reducing dimensional complexity, defined as:


$$Z_{h-e}^{\left(2\right)}=W_{h-e}^{\left(2\right)}\cdot A_{h-e}^{\left(1\right)}+b_{h-e}^{\left(2\right)}$$ (3)

$$A_{h-e}=\sigma (Z_{h-e}^{\left(2\right)})$$ (4)

Here, $W_{h-e}^{\left(2\right)}$ and $b_{h-e}^{\left(2\right)}$ denote the weight matrix and bias term of the second MLP layer, and $A_{h-e}$ is the final low-dimensional core feature representation.

This approach captures higher-order interaction effects through the MLP’s hidden-layer nonlinear transformations while using downscaling to mitigate the dimensionality catastrophe (Bengio et al., 2013; Wang et al., 2024). This enables more precise extraction of potential associations between human traits and environmental features (e.g., the moderating effect of narcissistic type on perceptions of social exclusion; Giacomin and Jordan, 2019). Meanwhile, after receiving data from the Narcissism Score Module and the Hostility Interpretation Module—processed through upscaling or downscaling—the model predicts whether the individual is in a state of social exclusion, and outputs this result alongside relevant data from these modules.

### 2.3 Narcissism score module

The results obtained by participants through the Explicit Narcissism Personality Scale and the Implicit Narcissism Personality Scale are entered into the Narcissism Score Module.

After collecting the data, we upscale it using a multilayer perceptron to improve the complexity and expressiveness of the narcissism scores. Let $X_{n}$ denote the original narcissism score data (e.g., scores from explicit and implicit narcissism scales). The upscaling process is formulated as:


$$Z_{n}^{\left(1\right)}=W_{n}^{\left(1\right)}\cdot X_{n}+b_{n}^{\left(1\right)}$$ (5)

$$A_{n}=\sigma (Z_{n}^{\left(1\right)})$$ (6)

Where $W_{n}^{\left(1\right)}$ and $b_{n}^{\left(1\right)}$ are the weight matrix and bias term of the MLP layer, respectively, and σ(·) is an activation function (e.g., ReLU) that introduces nonlinearity. $A_{n}$ represents the upscaled narcissism feature representation with enhanced complexity.

The upscaled narcissism data are then merged with data from the Human Characteristics and Environment Module. This feature fusion strategy is designed to retain as much interaction information between variables as possible by splicing feature vectors from different sources. For example, upscaled features of dominant narcissism scores may interact with environmental variables related to social exclusion to jointly predict hostile interpretations (Giacomin and Jordan, 2019). This data integration approach has been validated in psychological research, such as studies examining the relationship between narcissistic traits and structural and functional brain networks (Nenadić et al., 2021).

### 2.4 Hostile interpretation module

We first divided the hostile interpretation scores into two groups: one with scores ranging from 15 to 35 and the other with scores ranging from 36–55. This division is based on the actual distribution of scores (as shown in Table 1), clearly classifying “low-level hostile interpretation” (15–35) and “high-level hostile interpretation” (36–55) to facilitate subsequent analysis of differences across groups and situations. The Hostile Interpretation Module has two functions: (1) It can predict hostile interpretations using input from the Human Characteristics and Environment Module and the Narcissism Score Module. (2) It can use the same two modules to predict whether the person is currently in a socially exclusionary or socially accepting environment.

**Table 1:** Means and Standard Deviations of hostile interpretation scores and non-hostile interpretation scores of three groups of participants under different situations.

Situation type	Narcissism	Hostile interpretation score		Non-hostile interpretation score	
		M	SD	M	SD
Social exclusion	Neutral control group	30.09	6.65	42.50	7.40
	Dominant narcissism group	45.65	4.06	32.73	7.00
	Implicit narcissism group	43.91	4.95	34.82	4.68
Social acceptance	Neutral control group	30.56	6.53	42.53	7.16
	Dominant narcissism group	36.68	7.70	36.39	8.07
	Implicit narcissism group	43.64	4.55	34.69	6.19

M stands for mean, and SD stands for standard deviation.

Before feeding into the Hostile Interpretation Module, feature splicing is performed in two steps. First, the dimensionality-reduced feature $A_{h-e}$ from the Human Characteristics and Environment Module is concatenated with the upscaled feature $A_{n}$ from the Narcissism Score Module to form a comprehensive feature vector:


$$A_{h-e-n}=A_{h-e}⊕A_{n}$$ (7)

Note: The symbol ⊕ denotes feature concatenation (i.e., combining multiple feature vectors into a single comprehensive vector).

Then, for reverse prediction of the social exclusion, this concatenated feature is further spliced with the intermediate feature $A_{h,\mathrm{mid}}$ from the Hostile Interpretation Module itself, resulting in the input for reverse prediction:


$$A_{\mathrm{all}}=A_{h-e-n}⊕A_{h,\mathrm{mid}}$$ (8)

After receiving input data from the Human Characteristics and Environment Module and the Narcissism Score Module—via MLP-based upscaling or downscaling—the spliced data is processed through its own multilayer perceptrons, which serve two independent functions.

For the forward prediction of the environmental state, the spliced feature $A_{\mathrm{all}}$ is input into another one-layer MLP, and a Sigmoid function is used to output a binary classification of environmental state (social exclusion or acceptance):


$$Z_{e}^{\left(1\right)}=W_{e}^{\left(1\right)}\cdot A_{\mathrm{all}}+b_{e}^{\left(1\right)}$$ (9)

$$A_{e}=\sigma (Z_{e}^{\left(1\right)})$$ (10)

$${\hat{y}}_{e}=\sigma (W_{e}\cdot A_{e}+b_{e})$$ (11)

Where $W_{e}^{\left(1\right)}$ , $b_{e}^{\left(1\right)}$ and $W_{e}$ , $b_{e}$ are weight matrices and bias terms, and σ(·) here refers to the Sigmoid function.

For the reverse prediction of hostile interpretations, the concatenated feature $A_{h-e-n}$ is fed into a one-layer MLP to output a continuous hostile interpretation bias value:


$$Z_{h}^{\left(1\right)}=W_{h}^{\left(1\right)}\cdot A_{h-e-n}+b_{h}^{\left(1\right)}$$ (12)

$$A_{h}=\sigma (Z_{h}^{\left(1\right)})$$ (13)

$${\hat{y}}_{h}=W_{h}\cdot A_{h}+b_{h}$$ (14)

Where $W_{h}^{\left(1\right)}$ and $b_{h}^{\left(1\right)}$ are weight matrices and bias terms, and σ(·) is an activation function (e.g., ReLU).

### 2.5 Evaluation indicators

The evaluation metrics used in our model are as follows:

- Accuracy (ACC): Our HIBPN can predict hostile interpretation and to reverse-engineer social exclusion or social acceptance environments, so we use ACC to measure the predictive power of our model.
- F1 score: Considering that the ratio of data with the social exclusion condition or social acceptance condition is not 1:1, we use the F1 score to balance precision and recall. In category imbalance scenarios, the F1 score is effective in avoiding model overfitting for majority classes (e.g., social exclusion) and highlighting the ability to recognize minority classes (e.g., social acceptance) (Chawla et al., 2002).
- Receiver operating characteristic curve (ROC): Our model needs to invert the state of social exclusion or social acceptance through hostile interpretation—a dichotomous task—and the ROC curve gives a visual representation of the model’s ability to generalize under different decision thresholds (Fawcett, 2006). Its strength lies in the dynamic trade-off between the combined true-positive and false-positive rates, and it is particularly suitable for prediction tasks that require thresholds to be adjusted according to business scenarios (e.g., COVID-19 mortality prediction; Mendoza-Hernandez et al., 2024).
- Area under the ROC curve (AUC): Owing to the imbalance in the proportion of “social exclusion” and “social acceptance” data, ACC can be dominated by social exclusion results, which ultimately distorts the ACC. AUC more reliably assesses the model’s ability to discriminate between minority classes by integrating performance across all thresholds (Berrar et al., 2019). It complements the F1 score, which measures global performance from the perspective of ranking quality, and the F1 score, which assesses local performance from the perspective of the balance between precision and recall. For example, in social exclusion research, AUC has been used to evaluate models that predict social exclusion (Smith et al., 2023).

### 2.6 Comparison model

- Our model will be compared with the logistic regression, random forest, gradient boosting, AdaBoost, Support Vector Machine, Naive Bayes, Quadratic Discriminant Analysis, and decision tree models to evaluate ACC, F1 score, receiver operating characteristic curve, and area under the ROC curve.
- Logistic regression: Based on linear regression, it maps the output within the interval [0,1] using a Sigmoid function. This method serves as a benchmark for dichotomous classification tasks and is grounded in the generalized linear model framework (Nelder and Wedderburn, 1972), commonly used in psychological studies to predict dichotomous behavior (Dhariwal et al., 2024).
- Random forest: Based on the Bagging integration strategy, it constructs multiple decision trees and aggregates the final results through classification or regression. It has excellent stability and resistance to overfitting in high-dimensional data (Breiman, 2001).
- Gradient boosting: Based on the Boosting integration strategy, it iteratively trains weak learners to gradually improve overall performance by fitting the residuals of the previous model.
- AdaBoost: Trains weak classifiers iteratively, adjusting weights according to the errors of the previous model so that subsequent models pay more attention to difficult-to-classify samples. Ultimately, it combines all weak classifiers through weighted voting. It is theoretically based on the equivalence of “strongly learnable” and “weakly learnable” (Schapire, 1990).
- Support Vector Machine (SVM): Finds hyperplanes that maximize sample spacing for classification. Linearly inseparable data can be mapped to higher-dimensional spaces using a kernel function. The introduction of kernel methods has led to superior performance in nonlinear classification (Cortes and Vapnik, 1995).
- Naive Bayes: Based on Bayes’ theorem, it calculates the posterior probability that a sample belongs to each class and assigns the maximum value as the prediction. It is competitive in high-dimensional sparse data. For example, in text sentiment analysis, McCallum and Nigam (1998) conducted related research.
- Quadratic Discriminant Analysis (QDA): Calculates posterior probabilities and classifies data by estimating the mean vector and covariance matrix for each category. This method relaxes the linear classification assumption and is suitable for scenarios with heterogeneous covariance matrices (Friedman, 1989).
- Decision tree: Recursively divides the feature space and selects split nodes based on information gain, Gini index, etc., forming a tree structure. Its interpretability makes it a common tool for rule extraction (Quinlan, 1986).

## 3 Experiment 1: Measurement of hostile interpretation Bias

### 3.1 Experimental purpose

This experiment aimed to systematically collect indicators of participants’ dominant narcissism, implicit narcissism levels and hostile interpretation bias through social exclusion situational manipulation; incorporate the above-collected indicators into the HIBPN to verify the predictive relationships among narcissism (including dominant and implicit subtypes), social exclusion and hostile interpretation bias, clarify the specific manifestations of their interactions, and provide basic data support for subsequent exploration of the formation mechanism and intervention paths of hostile bias among narcissists.

### 3.2 Experimental methodology

#### 3.2.1 Participants

We used G*Power 3.1.9.7 software to estimate the required sample size in advance (Faul et al., 2007). At a significance level of *α* = 0.05 and a medium effect size (*f* = 0.25), a total sample size of at least 158 was required to achieve 80% statistical power. We distributed 315 copies of the Narcissistic Personality Scale to current undergraduate and graduate students at a university in Liaoning Province and a top-ranking university in Shandong province, China. A total of 258 valid questionnaires were returned, with an effective response rate of 81.9%. Using Excel, we ranked the Narcissistic Personality Scale scores from highest to lowest. The top 27% of students on the Implicit Narcissistic Personality Questionnaire were selected as the implicit narcissism group for the second part of the experiment. All participants were right-handed, had normal vision, were informed of the risks and benefits of the experiment, and participated voluntarily.

#### 3.2.2 Experimental materials

##### 3.2.2.1 Dominant narcissistic personality inventory

We used the Chinese version of the Narcissistic Personality Inventory developed by Wang (2008), based on the shortened version of the NPI for non-clinical populations (NPI-16) by Ames et al. (2006). The scale consists of 16 items scored from 0 to 16, with higher scores indicating greater levels of dominant narcissism. The scale’s internal consistency coefficient was 0.71, and the test–retest reliability was 0.72.

##### 3.2.2.2 Implicit narcissistic personality scale

We used the Hypersensitive Narcissism Scale to assess implicit narcissism. The scale contains 10 items on a unidimensional 5-point Likert scale ranging from 1 (“does not apply”) to 5 (“applies”), using the Chinese version revised by Wang (2008). The internal consistency coefficient was 0.73, and the test–retest reliability was 0.71, meeting psychometric standards.

##### 3.2.2.3 Hostile bias material

Interpretation bias materials were revised from those used by Kuckertz and Amir (2014) and Miao (2020). Thirty-two ambiguous sentences relevant to college students’ daily life (each with hostile and non-hostile interpretations) were initially selected. Fifteen psychology graduate students rated sentence familiarity, ambiguity, and interpretive validity. Based on these ratings, 24 sentences were chosen: 12 each for ambiguous and neutral (interference material). Two possible interpretations followed each sentence, and participants rated the likelihood of each using a 5-point Likert scale. For ambiguous sentences, one interpretation was hostile and the other non-hostile; both interpretations of neutral sentences were neutral.

##### 3.2.2.4 Environmental simulation: Cyberball passing game

The Cyberball 5.0 passing game was used to simulate social exclusion. Developed by Williams et al. (2000), Cyberball is a simple, interactive ball-passing game. Participants were told the experiment measured mental imagery skills and that they would be passing a ball online with two other players in different labs. Participants were instructed to vividly imagine the ball-passing scenario. The other two players were actually computer-generated avatars, whose information was masked to appear realistic. When the participant’s avatar received the ball, they used the mouse to choose which player to pass it to. A total of 30 passes were made, with the time between passes randomly varying from 0 to 4 s.

##### 3.2.2.5 Environmental simulation test scale: basic needs scale

The Basic Needs Scale consists of two parts. The first part measures the threat to basic needs, including four dimensions: belonging, self-esteem, existential significance, and sense of control. It includes 20 items with an internal consistency coefficient of 0.84. A 5-point Likert scale was used (1 = “not at all true” to 5 = “very true”), with items 1, 3, 5, 7, 9, 11, 14, 16, and 20 reverse-scored. Higher total scores indicate lower perceived threat to basic needs. The second part was a post-experiment questionnaire with three items. Participants subjectively reported their feelings of exclusion during the game (e.g., “Other players ignored me” and “Other players excluded me”) on a 5-point Likert scale. The third item asked participants to estimate their catch rate during the game, with their responses recorded in blank fields.

### 3.3 Experimental procedure

**Figure 3:**
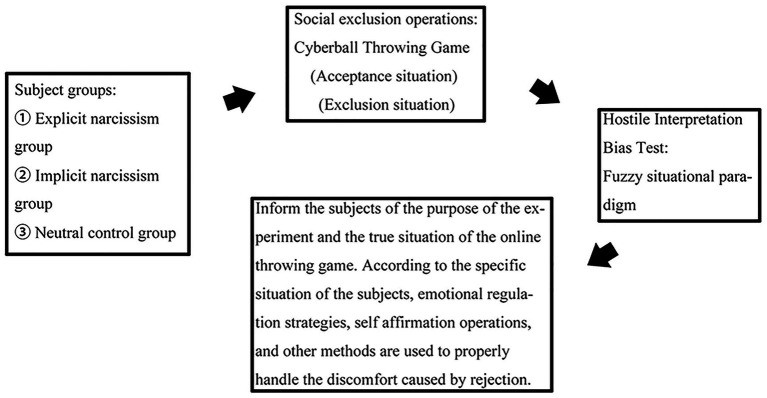
Experimental procedure 1 workflow: Subjects (explicit/implicit narcissism, neutral control groups) complete Cyberball social exclusion/acceptance, a fuzzy situational hostile interpretation bias test, then receive debriefing and emotional adjustment.

Figure 3 depicts the experimental flowchart.

First, participants independently completed the Dominant Narcissistic Personality Inventory and the Implicit Narcissistic Personality Scale in a quiet laboratory environment. Researchers collected their narcissistic trait data and used the top 27% of scores on the Implicit Narcissistic Personality Scale and the top 27% of scores on the Dominant Narcissistic Personality Inventory to categorize participants into the implicit narcissism group and dominant narcissism group, respectively; those with scores in the middle 46% were assigned to the neutral control group. Second, within each narcissistic trait group (dominant/implicit/neutral), participants were randomly assigned to either the social exclusion group or the social acceptance group using a random number table. They were informed that the experiment was a collaborative project with a university’s “Cognitive Laboratory” and would involve playing an online ball-passing game with two other “participants” (actually computer-generated avatars) via the Internet. They were instructed to vividly imagine the real ball-passing scenario, focus on the experimental process, and avoid discussing the task with others during the experiment. Third, the Cyberball task was implemented according to preset rules using the Cyberball 5.0 software: in the social exclusion group, participants received the ball only twice out of 30 total passes, and the avatars passed exclusively between themselves in the second half, resulting in a 6.67% pass rate for participants; in the social acceptance group, participants had a 33.33% probability of receiving the ball throughout the task. After completing the Cyberball game, participants immediately filled out the Basic Needs Scale to test the effectiveness of the social exclusion/acceptance manipulation, with no time limit for completion. Finally, under the researcher’s guidance, participants completed the Hostile Interpretation Bias Test using E-Prime 2. The test procedure included the presentation of a fixation point (“+”) for 500 ms, followed by the display of ambiguous contextual sentences or neutral interference sentences (each presented for 3,000 ms), and then participants rated the likelihood of two interpretations (1 = “extremely unlikely” to 5 = “extremely likely”)—hostile/non-hostile for ambiguous sentences, and both neutral for interference sentences. The presentation order of sentences and interpretations was randomized for each participant. After all tasks were completed, the researcher conducted a one-on-one debriefing, explaining the true purpose of the experiment to participants, and provided targeted emotion regulation strategies and self-affirmation exercises to alleviate psychological discomfort for those in the social exclusion group who reported a total score below 60 on the Basic Needs Scale (indicating high perceived threat to basic needs).

### 3.4 Result

**Figure 4:**
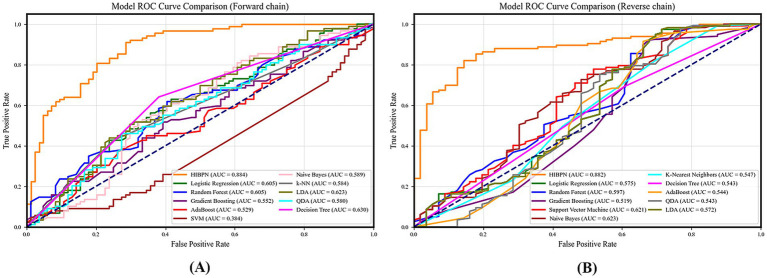
ROC curve comparison of HIBPN and traditional models. **(A)** Forward chain: prediction of social exclusion (AUC = 0.884). **(B)** Reverse chain: prediction of hostile interpretation (AUC = 0.882). HIBPN outperforms traditional methods in both chains.

**Figure 5:**
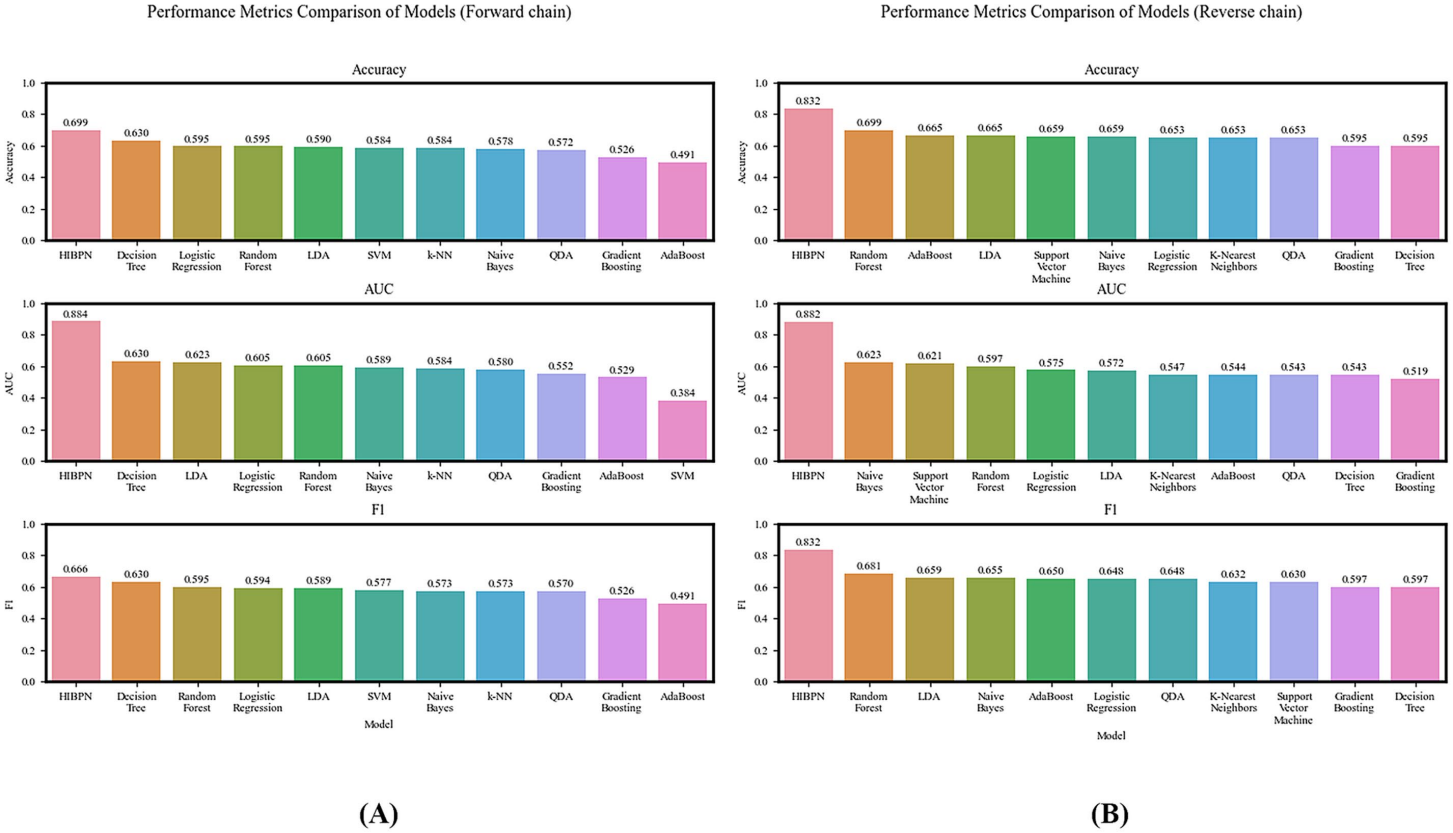
Model performance metrics (accuracy, AUC, F1 score) comparison: HIBPN outperforms traditional models in both forward **(A)** and reverse **(B)** chains.

**Figure 6:**
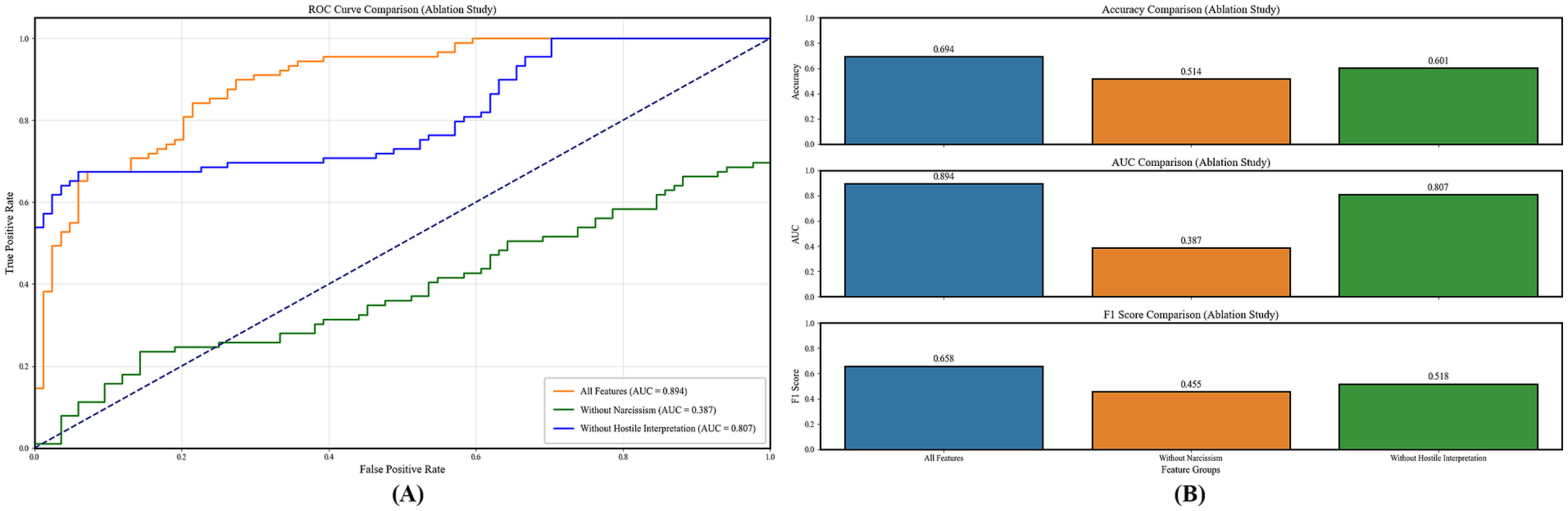
Ablation study results: Full-feature HIBPN (AUC = 0.894) outperforms models excluding narcissism/hostile interpretation in ROC curves **(A)** and metrics (Accuracy/AUC/F1) **(B)**.

We use Python 3.9 to run the code and compare the ACC, AUC, ROC, F1 scores of all the traditional machine learning methods mentioned above, as well as the HIBPN (see Figures 4–6 and Tables 2, 3).

**Table 2:** Comparative experimental results of the HIBPN two-way chaining with statistical methods and traditional machine learning methods under the ACC, AUC, and F1 score evaluation metrics.

Evaluation metrics	HIBPN	Logistic regression	Random forest	Gradient boosting	AdaBoost	SVM	Naive Bayes	k-NN	LDA	QDA	Decision tree
ACC (FC)	0.699^*^	0.595	0.595	0.526	0.491	0.584	0.578	0.584	0.590	0.572	0.630
ACC (RC)	0.832^*^	0.653	0.699	0.595	0.688	0.659	0.659	0.653	0.665	0.653	0.595
AUC (FC)	0.884^*^	0.605	0.605	0.552	0.529	0.384	0.589	0.584	0.623	0.580	0.630
AUC (RC)	0.832^*^	0.648	0.681	0.597	0.666	0.630	0.655	0.632	0.659	0.648	0.597
F1 score (FC)	0.666^*^	0.594	0.595	0.526	0.491	0.577	0.573	0.573	0.589	0.570	0.630
F1 score (RC)	0.882^*^	0.575	0.597	0.519	0.536	0.621	0.623	0.547	0.572	0.543	0.543

HIBPN = hostile interpretation and bidirectional prediction network, ACC = accuracy, AUC = area under the receiver operating characteristic (ROC) curve, FC = forward chain, RC = reverse chain. *The highest value among all evaluation metrics.

**Table 3:** Comparative experimental results under the ACC, AUC, and F1 score evaluation metrics for ablation experiments.

Evaluation metrics	All features	Without narcissism	Without hostile interpretation
ACC	0.694	0.514	0.601
AUC	0.894	0.387	0.807
F1 score	0.658	0.455	0.518

ACC = accuracy, AUC = area under the receiver operating characteristic (ROC) curve.

The ROC curve for the forward chain (core analysis of Experiment 1) shows that when classifying between social exclusion or social acceptance states, our HIBPN model achieves an AUC of 0.884. This indicates that, across different thresholds, our model can distinguish between these two states effectively. Among traditional machine learning methods, the decision tree, LDA, and random forest also show good performance, but a gap exists compared to the HIBPN model (the decision tree drops by about 28.73%, LDA by about 29.52%, and random forest and logistic regression by about 31.56%). Next are Naive Bayes and k-NN (down about 33.37 and 33.93%), followed by QDA, gradient boosting, and AdaBoost, which have AUCs ranging from 0.529 to 0.580. Gradient boosting and AdaBoost achieve AUCs of approximately 0.5, which is only slightly above the baseline level and close to random guessing. Finally, SVM has an AUC of 0.384, likely owing to underfitting when training with a 1:9 sample ratio.

In the forward chain prediction, the HIBPN model achieves the highest prediction ACC at 0.699. The decision tree also performs quite well at 0.630 (the highest among all traditional machine learning methods). Gradient boosting and AdaBoost achieve approximately 0.5 in ACC, which is no better than guessing. Meanwhile, our HIBPN achieves the highest F1 score of 0.666, indicating the best balance of precision and recall among the compared methods. This is followed by the decision tree, random forest, logistic regression, LDA, SVM, Naive Bayes, k-NN, QDA, gradient boosting, and AdaBoost methods, in this order. Gradient boosting and AdaBoost achieve F1 scores of approximately 0.5, which is not ideal.

In the reverse chain (supplementary analysis of Experiment 1), the HIBPN has the highest ACC, AUC, and F1 score, outperforming the best-performing traditional machine learning method by more than 15.98% across evaluation metrics. This demonstrates that our model is better at predicting the environments in which humans live under conditions of multifactor interactions. Our 1:9 training-to-test ratio further indicates the HIBPN’s strong generalization capability.

In the ablation experiments (auxiliary validation of Experiment 1), we found that removing key modules severely impairs the model’s prediction ability:

- Without the Hostile Interpretation Module: ACC drops by about 13.40%, AUC by about 9.73%, and F1 score by about 21.28%;
- Without the Narcissistic Score Module: ACC drops by about 35.02%, AUC by about 56.71%, and F1 score by about 30.85%.

#### 3.4.1 Core variable importance analysis

**Figure 7:**
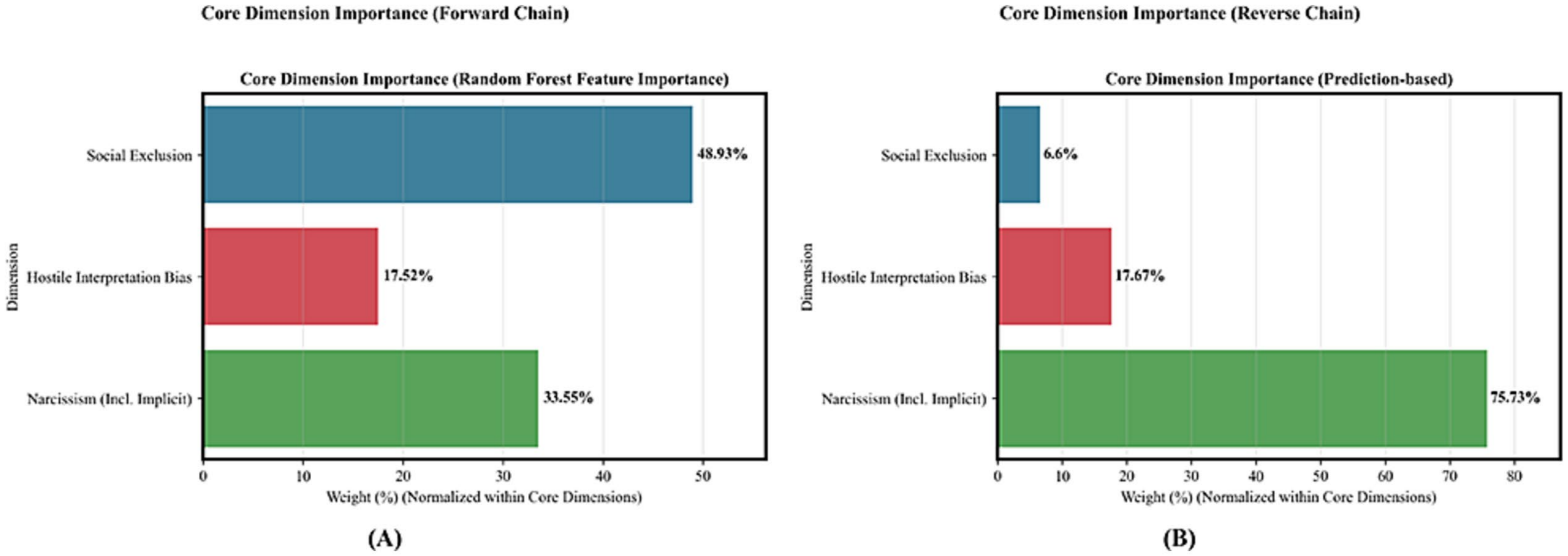
Core dimension importance distribution across bidirectional chains: Panel **(A)** (forward chain) shows narcissism as the second dominant factor, while panel **(B)** (reverse chain) indicates narcissism as the absolutely leading core dimension.

Due to the poor inherent interpretability of neural networks, we quantified the importance of the three core dimensions (social exclusion, hostile interpretation bias, and narcissism) using random forest feature importance scores, with a consistent methodology applied to both the forward and reverse chains (see Figure 7 and Table 4).

**Table 4:** Core dimension importance weights across bidirectional prediction chains.

Core dimension	Forward chain weight (%)	Reverse chain weight (%)
Social exclusion	48.93	6.6
Hostile interpretation bias	17.52	17.67
Narcissism (incl. implicit)	33.55	75.73

Figure 7 intuitively illustrates the contribution disparities of core dimensions across the bidirectional prediction chains: In the forward chain (Panel A), the weight of the narcissism dimension (including implicit narcissism) (33.55%) is significantly higher than that of hostile interpretation bias (17.52%), making it the second most prominent driving factor only after social exclusion. In the reverse chain (Panel B), the weight of the narcissism dimension further rises to 75.73%, emerging as the absolutely dominant core variable.

This result is consistent with our expectation that narcissism is the core driving factor in the bidirectional prediction chains—whether predicting social exclusion states or reversely inferring hostile interpretation bias, narcissism occupies a critical position in the model’s decision-making logic. Meanwhile, this aligns with the findings of the ablation experiments: the substantial performance degradation of the model after removing the narcissistic module essentially stems from the core role of the narcissism dimension in feature contributions.

### 3.5 Discussion

#### 3.5.1 Interpretation of main findings

Experiment 1 validated the bidirectional prediction mechanism among narcissism, social exclusion, and hostile interpretation bias using HIBPN. Three core findings emerged.

First, HIBPN substantially outperformed all traditional machine learning methods. In the forward chain (predicting social exclusion status), HIBPN achieved an AUC of 0.884, ACC of 0.699, and F1 of 0.666—significantly exceeding the best traditional method (decision tree: AUC = 0.630). This validates dynamic systems theory’s core proposition (Thelen and Smith, 1994a, 1994b): cognitive states and social environments are bidirectionally coupled. HIBPN’s multilayer architecture with nonlinear activation functions successfully operationalized this “state-context dynamic equilibrium,” capturing complex interactions that linear models cannot represent.

Second, traditional methods show clear limitations. Even the best-performing traditional method (decision tree: AUC = 0.630) lagged 28.73% behind HIBPN; most methods (LDA, logistic regression, random forest) scored AUCs between 0.605 and 0.623, while Naive Bayes, k-NN, QDA, gradient boosting, AdaBoost, and SVM fell below 0.6 or approached random guessing. These data confirm that traditional statistical methods, constrained by linear additivity assumptions (Cohen et al., 2003; Pearl, 2009), and traditional machine learning methods, reliant on manual feature engineering and lacking bidirectional feedback mechanisms (Hastie et al., 2009; Goodfellow et al., 2016), cannot capture the complex interactions among narcissism, hostile interpretations, and social exclusion.

Third, HIBPN’s bidirectional design operationalized “cognition-environment bidirectional coupling.” In the reverse chain (predicting hostile interpretation bias), HIBPN exceeded the best traditional method by >15.98% across all metrics. This confirms a bidirectional cycle: the forward path (“cognition → environment”: narcissism and hostile interpretations jointly predict social exclusion), and the reverse path (“environment → cognition”: narcissism and social exclusion jointly predict hostile interpretation bias). These findings extend Zeigler-Hill et al.'s (2008) “threat perception → hostile interpretation” chain into a complete bidirectional model.

#### 3.5.2 Core driving variable: cross-validation of ablation and feature importance analyses

Two complementary approaches identified narcissism as the core driver in this interaction system.

Ablation experiments revealed asymmetric module importance: removing the Hostile Interpretation Module reduced ACC by 13.40%, AUC by 9.73%, and F1 by 21.28%; removing the Narcissistic Score Module caused more dramatic declines—ACC down 35.02%, AUC down 56.71%, and F1 down 30.85%. Thus, the Narcissistic Score Module is irreplaceable.

Random forest feature importance analysis cross-validated this finding. In the forward chain, narcissism weight (33.55%) significantly exceeded hostile interpretation (17.52%), ranking second only to social exclusion. In the reverse chain, narcissism weight surged to 75.73%, becoming absolutely dominant. Together, these dual lines of evidence confirm narcissism as the core driver, with influence far exceeding hostile interpretation.

This aligns with the Dualistic Model of Narcissism (Wink, 1991). Descriptive statistics (Table 1) show implicit narcissists scored highest on hostile interpretation (*M* = 43.16), followed by grandiose narcissists (*M* = 33.27) and controls (*M* = 30.12). This subtype-specific effect reflects implicit narcissists’ dependence on external validation and hypersensitivity to social threats (Zeigler-Hill et al., 2008; Miller et al., 2011), versus grandiose narcissists’ defensive reactions when superiority is threatened (Pincus et al., 2014; Lamkin et al., 2014). This cognitive mechanism, not explicitly articulated in the original model, receives empirical support here.

#### 3.5.3 Methodological necessity of deep learning

A key contribution is demonstrating that deep learning is not merely an alternative but a methodological necessity for this research question.

As shown in Section 3.5.1, traditional methods performed near chance (AUC < 0.6) while HIBPN achieved superior performance (AUC = 0.884). This gap confirms that the complexity of underlying mechanisms—higher-order nonlinear interactions and bidirectional feedback—exceeds traditional methods’ representational capacity.

Traditional statistical methods cannot model synergistic effects (e.g., implicit narcissism × low belongingness) without pre-specified interaction terms. Traditional machine learning methods, though handling some nonlinearity, require manual feature engineering and lack architectural flexibility for bidirectional feedback, preventing simultaneous validation of both “cognition → environment” and “environment → cognition” directions.

Deep learning provides two critical advantages. First, multilayer architectures with nonlinear activation functions automatically learn high-order feature interactions. Second, HIBPN’s bidirectional design explicitly operationalizes dynamic systems theory, capturing both forward and reverse paths simultaneously. Combined with strong generalization under 1:9 training-test splitting, these features establish deep learning as necessary for modeling dynamic, bidirectional relationships among narcissism, hostile interpretations, and social exclusion.

#### 3.5.4 Limitations and future directions

Experiment 1 has four main limitations.

First, sample representativeness. Participants were undergraduate and graduate students from Liaoning and Shandong Provinces (ages 19–22), limiting generalizability. Future research should include diverse ages, occupations, and cultural backgrounds to test cross-population stability.

Second, model interpretability. As a deep learning model, HIBPN has “black box” limitations. Although random forest feature importance partially compensated, this remains post-hoc. Future research should integrate interpretable AI methods (e.g., SHAP, LIME) to clarify psychological pathways.

Third, research paradigm. The Cyberball paradigm, though validated, has limited ecological validity. Future research should use more naturalistic manipulations (e.g., interpersonal conflict, real-world exclusion) to test HIBPN’s robustness across contexts.

Fourth, narcissism subtype coverage. This study examined grandiose and implicit narcissism only. Future research should include other subtypes (e.g., collective, pathological narcissism) for a more comprehensive theoretical account.

## 4 Experiment 2: The effect of self-affirmation on hostile interpretation Bias in individuals with implicit narcissism under social exclusion

### 4.1 Experimental purpose

To investigate the intervention effect of preventive self-affirmation on hostile interpretation bias among individuals with implicit narcissism under social exclusion. Specifically, this experiment aims to verify whether preventive self-affirmation can effectively mitigate hostile interpretation bias in implicit narcissists and enhance their predicted rate of social acceptance in social interactions.

### 4.2 Experimental methodology

#### 4.2.1 Participants

We also used G*Power 3.1.9.7 to estimate the required sample size *a priori* (Faul et al., 2007). At a significance level of *α* = 0.05 and a medium effect size (*f* = 0.25), the required total sample size for 80% statistical power was at least 34. A total of 36 valid participants were recruited from a top-ranking university in Shandong province, China, aged 19–22 years (M-20.42, SD = 1.05), including 24 men and 12 women. All participants were right-handed, had normal vision, were informed of the risks and benefits of the experiment, and participated voluntarily.

#### 4.2.2 Experimental materials

##### 4.2.2.1 Implicit narcissistic personality scale

The Implicit Narcissistic Personality Scale used in this experiment was the same as that in Experiment 1.

##### 4.2.2.2 Hostile bias material

The Hostile bias material was consistent with that used in Experiment 1.

##### 4.2.2.3 Environmental simulation: Cyberball passing game

The Cyberball passing game used to simulate social exclusion in this experiment was the same as that in Experiment 1.

##### 4.2.2.4 Environmental simulation test scale: basic needs scale

The Basic Needs Scale used in this experiment was identical to that in Experiment 1.

##### 4.2.2.5 Self-affirmation manipulation

In this study, self-affirmation was manipulated using a combination of values-based self-affirmation and trait-based self-affirmation. First, participants were asked to write down three good qualities they believed they possessed. Second, they were given a list of 13 values (e.g., money, career success, and academic achievement) and asked to rank them in order of personal importance from 1 (most important) to 13 (least important), with each number used only once. Next, participants were asked to write down why the top-ranked value was important to them and to provide a detailed example to illustrate it. Finally, they completed two questions using a five-point Likert scale. The first question was: “After doing the above, I would think of the positive aspects of myself.” The second was: “After doing the above, I would think of the things that are more important to me.” The scale ranged from 1 (“very inconsistent”) to 5 (“very consistent”). In the control group, participants were asked instead to recall and list all the food they had eaten in the past 48 h, specifying the time or type of food.

### 4.3 Experimental procedure

**Figure 8:**
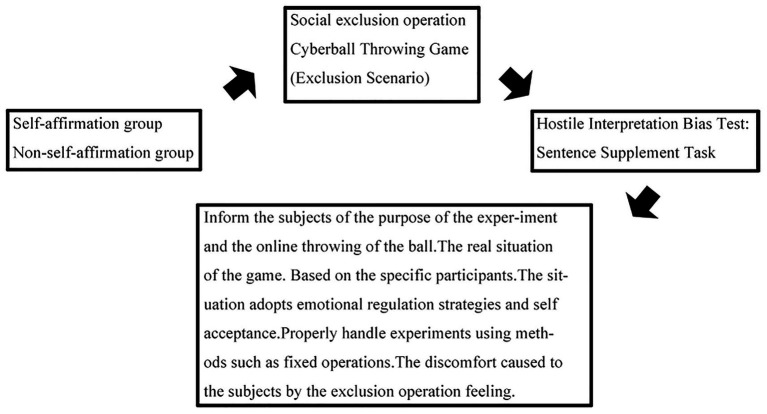
Experimental procedure 2 workflow: Self-affirmation/non-self-affirmation groups complete Cyberball social exclusion, a sentence-supplement hostile interpretation bias test, then receive debriefing and emotional adjustment.

Figure 8 depicts the experimental flowchart.

First, participants were randomly divided into the self-affirmation group and the control group using a random number table, with 18 participants in each group. Those in the self-affirmation group completed a combined values-based and trait-based self-affirmation task within 15 min: (1) wrote down three of their own good qualities; (2) ranked 13 core values (e.g., money, career success, and academic achievement) by personal importance; (3) explained the importance of the top-ranked value in 100–150 words and provided a detailed real-life example. Participants in the control group were asked to recall and list all food they had eaten in the past 48 h, specifying the exact time (e.g., 7:30 a.m. on Day 1) and type of food (e.g., steamed bun, milk), with the same 15-min time limit. Second, all participants completed the Implicit Narcissistic Personality Scale to confirm their implicit narcissistic traits, ensuring all participants met the “top 27% of scores” criterion from Experiment 1. Immediately after this, the self-affirmation group completed two 5-point Likert scale items to assess the effectiveness of the self-affirmation manipulation, while the control group proceeded directly to the next task without additional measures. Third, the assignment and implementation of the Cyberball task were consistent with Experiment 1: participants were randomly assigned to the social exclusion group or the social acceptance group using a random number table, with the same task rules (30 total passes, 6.67% pass rate for the exclusion group, 33.33% for the acceptance group) and operational procedures. Fourth, after the Cyberball task, participants completed the Basic Needs Scale to verify the effectiveness of the social exclusion/acceptance manipulation, with responses collected anonymously to ensure authenticity. Fifth, the Hostile Interpretation Bias Test was identical to that in Experiment 1 in terms of test tools, presentation format (fixation point duration, sentence display time), rating scale, and randomization rules, ensuring consistency in the measurement of hostile interpretation bias. Finally, referring to the process of Experiment 1, the researcher conducted a one-on-one debriefing to explain the true purpose of the experiment, answered any questions raised by participants, and provided emotional support to those who experienced negative emotions (e.g., feelings of exclusion, frustration), ensuring no adverse psychological impacts before participants left the laboratory.

### 4.4 Result

**Figure 9:**
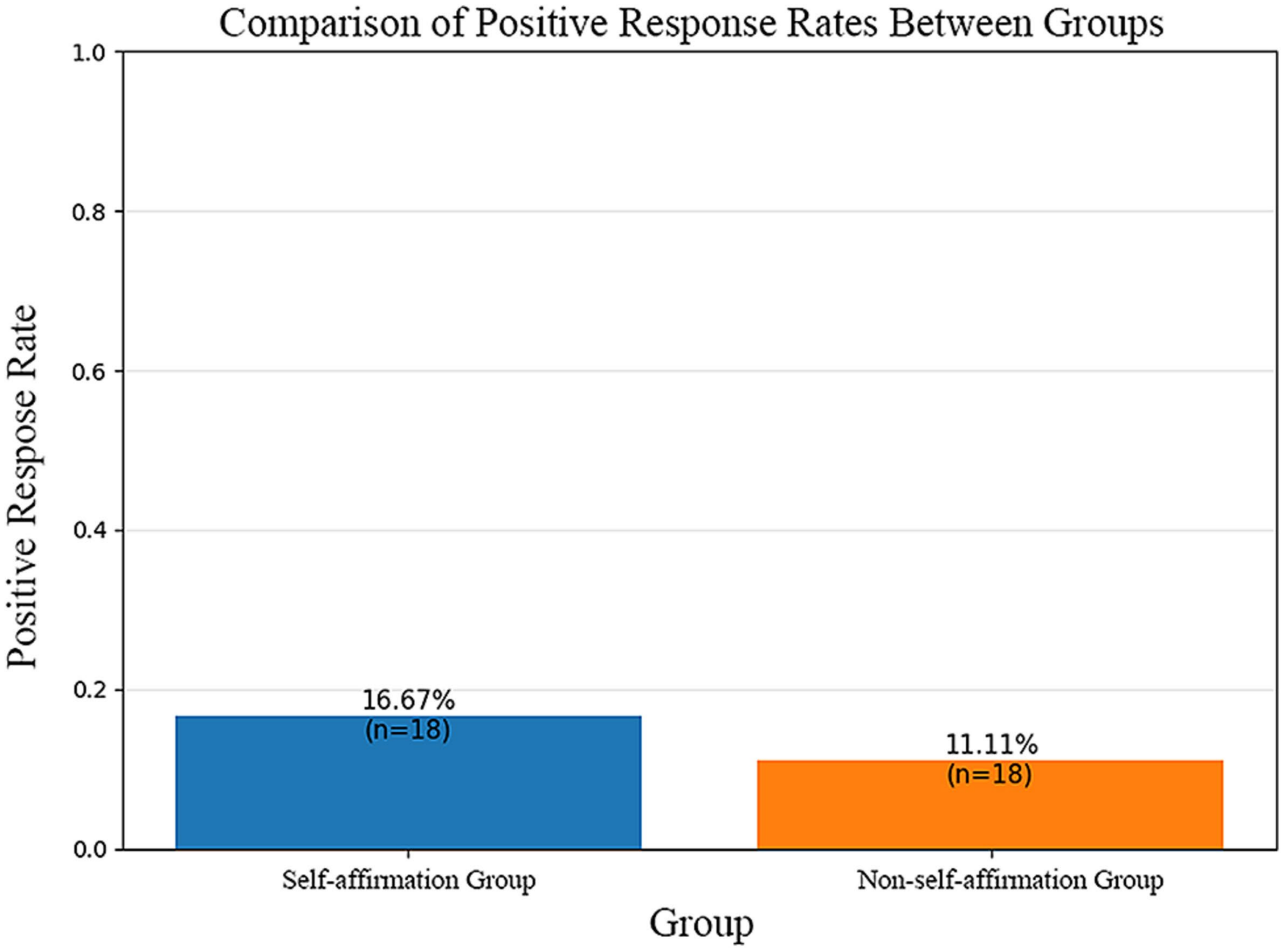
Intervention experiment results: The self-affirmation group (16.67%, *n* = 18) had a higher positive response rate than the non-self-affirmation group (11.11%, *n* = 18), aligning with reduced hostile interpretation bias in implicit narcissists.

Experiment 2 focuses on the intervention effect of self-affirmation, with the HIBPN model maintaining consistent performance (aligned with Experiment 1’s methodological framework) while quantifying the intervention outcome (as shown in the Figure 9):

The self-affirmation group was predicted to have a 5.56% higher social acceptance rate than the non-self-affirmation group. This indicates that preventive self-affirmation effectively mitigates hostile interpretation bias among implicit narcissists, improving their perceived social acceptance (see Tables 1–5).

**Table 5:** Prediction of social exclusion or social acceptance in self-affirming versus non-self-affirming groups under the HIBPN intervention experiment.

Evaluation metrics	Self-affirmation group	Non-self-affirmation group
Positive response rate	0.1667	0.1111

HIBPN = hostile interpretation and bidirectional prediction network.

### 4.5 Discussion

#### 4.5.1 Interpretation of main findings

Experiment 2 investigated the intervention effect of preventive self-affirmation on hostile interpretation bias in individuals with implicit narcissism under social exclusion. The results revealed that the self-affirmation group had a 5.56% higher predicted social acceptance rate compared to the non-self-affirmation group, indicating that preventive self-affirmation effectively mitigates hostile interpretation bias among implicit narcissists and improves their perception of social acceptance. This finding carries several important implications.

First, this result provides empirical support for the plasticity of cognitive biases in implicit narcissists. As demonstrated in Experiment 1, implicit narcissists exhibit significantly higher hostile interpretation bias (*M* = 43.16) compared to grandiose narcissists (*M* = 33.27) and controls (*M* = 30.12), a pattern attributed to their heightened dependence on external validation and hypersensitivity to social threats (Zeigler-Hill et al., 2008; Miller et al., 2011). The finding that a brief self-affirmation intervention (approximately 15 min) can significantly reduce this bias suggests that implicit narcissists’ hostile interpretive tendencies, while pronounced, are not immutable. This aligns with cognitive plasticity perspectives within social cognitive theory (Bandura and National Institute of Mental Health, 1986), which emphasize that cognitive patterns can be modified through self-regulatory processes.

Second, the findings extend our understanding of self-affirmation’s psychological mechanisms. Traditional self-affirmation theory posits that affirmation bolsters self-integrity, reducing defensive responses to threats (Steele, 1988; Sherman and Cohen, 2006). In the context of implicit narcissism, social exclusion poses a dual threat—it not only challenges belongingness needs but also threatens the fragile self-worth characteristic of vulnerable narcissism. Self-affirmation likely operates by reinforcing implicit narcissists’ sense of self-worth independent of external feedback, thereby reducing their tendency to interpret ambiguous social cues as hostile. This interpretation is consistent with the significant reduction in hostile interpretation bias observed in the self-affirmation group.

Third, the results provide intervention-level cross-validation of the bidirectional pathways revealed by HIBPN. Experiment 1 demonstrated that the relationship between hostile interpretations and social exclusion perceptions is bidirectional, with both forward path (cognition → environment) and reverse path (environment → cognition) showing significant predictive power. Experiment 2’s finding that reducing hostile interpretations (via self-affirmation) leads to improved social acceptance perceptions provides causal evidence for the reverse pathway. Specifically, the 5.56% increase in predicted social acceptance rate among the self-affirmation group indicates that modifying cognitive biases can indeed alter environmental perceptions, supporting the feedback reinforcement process identified in HIBPN’s reverse chain.

#### 4.5.2 Integration with HIBPN’S predictive framework

Experiment 2’s findings are particularly meaningful when viewed through HIBPN’s analytical lens. Within HIBPN’s bidirectional framework, the intervention effect can be understood as a targeted modulation of the reverse pathway:

Reverse pathway baseline: In Experiment 1, HIBPN’s reverse chain demonstrated that implicit narcissism and social exclusion jointly predict hostile interpretation bias, with narcissism accounting for 75.73% of feature importance—confirming its role as the core driver in this direction.

Intervention effect: Experiment 2 shows that modifying the cognitive outcome (hostile interpretation bias) through self-affirmation feeds back to alter environmental perception (social acceptance rate), confirming the bidirectional nature of this system. This finding aligns with Bandura and National Institute of Mental Health’s (1986) triadic reciprocal determinism, demonstrating that changes in cognitive-affective factors can influence both behavioral-cognitive outcomes and environmental perceptions.

Notably, the 5.56% improvement in predicted social acceptance rate, while modest in absolute terms, represents a meaningful shift when considering the stability of implicit narcissists’ cognitive patterns. This effect size is consistent with meta-analytic findings on self-affirmation interventions in clinical and social psychology (Epton et al., 2015), suggesting that brief, low-intensity interventions can produce measurable changes in cognitive biases.

#### 4.5.3 Theoretical implications

Experiment 2’s findings contribute to theoretical development in three key areas:

First, extending the Dualistic Model of Narcissism (Wink, 1991). While previous research has documented that implicit narcissists are more prone to hostile interpretations (Zeigler-Hill et al., 2008), few studies have explored intervention strategies targeting this bias. Experiment 2 demonstrates that self-affirmation—a relatively simple psychological intervention—can effectively reduce hostile interpretation bias in this population. This extends the Dualistic Model by suggesting that implicit narcissists’ cognitive vulnerabilities, while stable at the trait level, remain responsive to targeted interventions at the state level.

Second, enriching social cognitive theory (Bandura and National Institute of Mental Health, 1986). The finding that modifying cognitive biases (hostile interpretations) alters environmental perceptions (social acceptance rate) provides empirical support for the reciprocal determinism framework. Within Bandura’s triadic model, personal factors (narcissism), cognitive processes (hostile interpretations), and environmental factors (social exclusion) are understood to dynamically interact. Experiment 2’s demonstration that intervening at the cognitive level can shift environmental perceptions validates this bidirectional logic and complements Experiment 1’s correlational findings with causal evidence.

Third, bridging self-affirmation theory with narcissism research. Previous self-affirmation research has primarily focused on threat reduction in general populations (Sherman and Cohen, 2006) or health behavior change (Epton et al., 2015). Experiment 2 extends this literature by demonstrating self-affirmation’s effectiveness in addressing cognitive biases associated with vulnerable narcissism—a population characterized by heightened defensiveness and threat sensitivity. This suggests that self-affirmation may be particularly valuable for interventions targeting individuals with fragile self-worth.

#### 4.5.4 Clinical and practical implications

The findings of Experiment 2 carry important implications for intervention development:

First, preventive self-affirmation may serve as an accessible, low-intensity intervention for individuals with implicit narcissistic traits who are at risk for hostile interpretation biases. The brief, 15-min intervention used in this study could be easily adapted for educational settings, counseling centers, or online mental health platforms.

Second, the results suggest that targeting cognitive biases directly may be more efficient than attempting to modify environmental factors, particularly when working with populations characterized by stable personality traits. The 5.56% improvement in social acceptance perception was achieved through a single, brief intervention, highlighting the potential efficiency of cognitive-level interventions.

Third, the findings underscore the importance of early intervention. Given that Experiment 1 established implicit narcissism as the core driver in the bidirectional system (75.73% feature importance in reverse chain), interventions targeting cognitive biases in this population before they become entrenched may be particularly effective.

#### 4.5.5 Limitations and future directions

Experiment 2 has several limitations that warrant consideration:

First, sample size and composition. The sample of 36 participants (18 per group), while adequately powered based on *a priori* calculations (G*Power: minimum 34), remains relatively small. Moreover, participants were recruited from a single top-ranking university in Shandong Province, limiting generalizability to other populations. Future research should replicate these findings with larger, more diverse samples, including community samples and clinical populations with more pronounced narcissistic traits.

Second, single-session intervention. This study examined the immediate effects of a single self-affirmation session. Whether these effects persist over time remains unknown. Future research should incorporate follow-up assessments (e.g., 1 week, 1 month, 3 months) to examine the durability of the intervention effect and determine whether booster sessions might be beneficial.

Third, mechanism exploration. While this study demonstrates that self-affirmation reduces hostile interpretation bias, the precise psychological mechanisms remain unclear. Self-affirmation may operate through multiple pathways: bolstering self-integrity, reducing threat vigilance, enhancing cognitive flexibility, or a combination of these processes. Future research should include process measures to disentangle these potential mechanisms.

Fourth, generalizability to other contexts and outcomes. This study focused specifically on social exclusion induced by the Cyberball paradigm and measured hostile interpretation bias using ambiguous sentence materials. Whether the intervention effect generalizes to other forms of social threat (e.g., interpersonal conflict, romantic rejection) or other cognitive biases (e.g., attributional style, rumination) requires further investigation.

Fifth, comparison with other intervention approaches. This study did not compare self-affirmation with alternative interventions (e.g., cognitive reappraisal training, mindfulness, acceptance-based approaches). Future research should include active comparison conditions to establish the relative efficacy of self-affirmation for this population and identify potential moderators of treatment response.

Sixth, integration with HIBPN’s predictive capabilities. While Experiment 2 quantified the intervention effect as a 5.56% increase in predicted social acceptance rate, future research could leverage HIBPN’s bidirectional architecture to develop personalized intervention predictions. For example, HIBPN could potentially predict which individuals are most likely to benefit from self-affirmation based on their baseline characteristics, enabling targeted intervention allocation.

Despite these limitations, Experiment 2 provides promising evidence that preventive self-affirmation can effectively mitigate hostile interpretation bias in implicit narcissists under social exclusion. These findings, combined with Experiment 1’s demonstration of HIBPN’s predictive capabilities, lay important groundwork for understanding and addressing the cognitive mechanisms underlying aggression and social dysfunction in vulnerable populations.

## 5 Conclusion

This study developed the Hostile Interpretation and Bidirectional Prediction Network (HIBPN) to explore the dynamic interactions among narcissism, hostile interpretations, and social exclusion. Results showed that the model effectively captured the nonlinear and reciprocal relationships among the three variables, with bidirectional prediction performance outperforming traditional statistical methods and classic machine learning algorithms. Dual evidence from ablation experiments and random forest analysis confirmed that narcissism serves as the core driving variable in this interaction system. Additionally, implicit narcissism exhibited a stronger association with hostile interpretation bias, and preventive self-affirmation could effectively mitigate such bias.

Integrating core perspectives from social cognitive theory, dynamic systems theory, and the Dualistic Model of Narcissism, this study provides a new methodological template for modeling complex psychological mechanisms, identifies the feasible value of self-affirmation strategies in reducing hostile interpretations induced by social exclusion, and bridges deep learning innovation with psychological theories, laying a foundation for subsequent research on aggressive cognition and the development of targeted interventions.

## Data Availability

The original contributions presented in the study are included in the article/supplementary material, further inquiries can be directed to the corresponding author/s.
